# Pharmacokinetics of Pimobendan and Its Metabolite O-Desmethyl-Pimobendan Following Rectal Administration to Healthy Dogs

**DOI:** 10.3389/fvets.2020.00423

**Published:** 2020-08-04

**Authors:** Jiwoong Her, Kendon W. Kuo, Randolph L. Winter, Crisanta Cruz-Espindola, Lenore M. Bacek, Dawn M. Boothe

**Affiliations:** ^1^Department of Clinical Sciences, College of Veterinary Medicine, Auburn University, Auburn, AL, United States; ^2^Department of Anatomy, Physiology, and Pharmacology, College of Veterinary Medicine, Auburn University, Auburn, AL, United States

**Keywords:** congestive heart failure, rectal drug administration, pharmacokinetics, pimobendan, bioavailability

## Abstract

**Objective:** This study describes the pharmacokinetics of parent pimobendan (PIM) and its active metabolite, o-desmethyl-pimobendan (ODMP), after oral and rectal administration of pimobendan to healthy dogs.

**Animals:** A total of eight healthy privately owned dogs were used in this study.

**Procedures:** The dogs received a single dose (0.5 mg/kg) of a commercially available pimobendan tablet per os (PO). Twelve blood samples were collected over a 12-h period for pharmacokinetic analysis. After a 24-h washout period, the dogs received the same dose of pimobendan solution per rectum (PR), and samples were obtained at the same time for analysis.

**Results:** For PIM, PO vs. PR, respectively, the mean maximum plasma concentration (*C*_max_, ng/ml) was 49.1 ± 28.7 vs. 10.1 ± 2, the time to reach a maximum concentration (*T*_max_, h) was 2.1 ± 0.9 vs. 1 ± 0.4, the disappearance half-life (*t*_1/2_, h) was 1.8 ± 0.8 vs. 2.2 ± 0.6, and the area under the concentration–time curve (AUC, ng^*^h/ml) was 148.4 ± 71.6 vs. 31.1 ± 11.9, with relative bioavailability (*F*, %) of 25 ± 8. For ODMP, PO vs. PR, respectively, *C*_max_ was 30.9 ± 10.4 vs. 8.8 ± 4.8, *T*_max_ was 3.2 ± 1.6 vs. 1.7 ± 1.1, and *t*_1/2_ was 5.0 ± 2.7 vs. 8.3 ± 4.8, with AUC of 167.8 ± 36.2 vs. 50.1 ± 19.2 and *F* of 28 ± 6. The differences between PO and PR were significant (*P* < 0.03) for AUC and *C*_max_ for both PIM and ODMP.

**Conclusions and Clinical Relevance:** The pharmacokinetics of PIM and ODMP were described following PO and PR administration. The findings suggest that pimobendan PR might achieve effective concentrations and, as such, warrant future studies of clinical effectiveness in treating dogs with congestive heart failure and which are unable to receive medication PO.

## Introduction

Pimobendan, a benzimidazole-pyridazinone derivative, is known as an inodilator that possesses the unique combination of a positive inotrope and a vasodilator (1). It acts as a positive inotrope by sensitizing the affinity of calcium for binding to troponin C on cardiac myocytes and inhibiting phosphodiesterase 3 (PDE3). The inhibition of PDE3 also results in both arterial dilation and venodilation. As a result, pimobendan improves the cardiac output without increasing the myocardial oxygen consumption (1–3). This unique combination of properties makes pimobendan desirable in the treatment of congestive heart failure (CHF) secondary to myxomatous mitral valve disease or dilated cardiomyopathy in dogs (2–5). The American College of Veterinary Internal Medicine (ACVIM) Specialty of Cardiology recommends pimobendan use for acute, hospital-based therapy of patients with current clinical signs of CHF as well as for patients with advanced myxomatous mitral valve disease prior to the onset of CHF (ACVIM heart disease stages B2, C, and D) (4).

While the oral bioavailability of the commercial tablet is 70% (1, 2, 6), oral administration is not a viable route in an emergency situation. Many dogs presenting with advanced stage of CHF are in severe respiratory distress on presentation. Furthermore, some dogs are very fearful or otherwise difficult to medicate *per os*. As such, oral administration may not be possible. For those scenarios, injectable pimobendan has been developed and used in multiple countries including Australia, Japan, and the United Kingdom. When given intravenously, injectable pimobendan provides a rapid inotropic effect and decreases left ventricular end diastolic pressure in healthy dogs (7). However, to date, pimobendan is currently commercially available only as an oral, chewable tablet (Vetmedin, Boehringer-Ingelheim, Ingelheim, Germany) in other countries including the United States. The manufacturer does not anticipate pursuing an approval of any other pimobendan formulation in the United States. The lack of availability of an alternative route of pimobendan limits its use in dogs in those countries where the injectable formulation is not available.

Studies have shown that rectal administration can be a viable option for dogs who cannot receive medications per os (PO) (8–11). The potential advantages of rectal administration compared to oral administration include less stress for the patient, more rapid absorption, and decreased risk to the administrator in the case of patients who are otherwise unable to take oral medication (12, 13). Due to the lack of injectable pimobendan, practitioners in the United States and other countries have anecdotally administered pimobendan per rectum (PR) in emergencies. To the authors' knowledge, the rectal administration of pimobendan to dogs with CHF is supported only by anecdotal reports. Therefore, the efficacy or the appropriate dose of pimobendan for rectal administration to dogs is unknown. In addition, there is sparse data regarding the pharmacokinetics of pimobendan's active metabolite o-desmethyl-pimobendan (ODMP; UD-CG 212 Cl) in dogs, despite findings which suggest that ODMP may be a significant contributor to the hemodynamic effects of pimobendan (2, 14).

The purpose of this study was to describe the pharmacokinetic characteristics of parent pimobendan (PIM) and ODMP following a single dose (0.5 mg/kg) of oral or rectal administration in healthy dogs to explore the prospect of its clinical application. Our goal was to identify a therapeutic dose of pimobendan PR for dogs. This study aimed to obtain the relative bioavailability with a hypothesis that when pimobendan is administered rectally, pimobendan and ODMP will achieve plasma concentrations previously demonstrated to be therapeutic based on orally administered drug and that the increase in dose necessary to increase these concentrations will be <3-fold of the oral dose (15). Finally, we compared the pharmacokinetics of pimobendan following rectal administration as determined in this study to those following oral administration as determined in previous studies and as per the package insert (15) to determine whether rectal administration might be an appropriate route for dogs in which oral administration of medications is limited.

## Materials and Methods

### Animals

Eight privately owned healthy adult dogs (three males and five females) were included in the study. The sample size was designed to be able to consider a 40% difference in area of the curve being significantly different between the two routes (PO and PR), given a variability of 40% around the mean difference. Based on the maximum plasma concentration (*C*_max_) and 90% confidence level, it was determined that eight dogs were necessary to describe a confidence interval that ranged 30% about the mean. The dogs included in the study were 3.7 ± 2.5 years of age and weighing 26.8 ± 5.8 kg. All dogs were determined to be healthy based on benign medical history, normal physical examination, systolic blood pressure measured via Doppler technique, bloodwork (complete blood count and chemistry panel), and urinalysis. All dogs did not receive any drugs prior to and during the experiments other than the preventive medication. During the study period, all the dogs were housed individually in a ward designated for research within a veterinary teaching hospital. All the dogs were observed for adverse effects for a period of 12 h after drug administration and daily throughout the study period. All study protocols were approved by the institutional laboratory animal care and use committee, and informed consent was obtained from all the owners before enrollment into the study.

### Experimental Design and Drug Administration

The dogs were studied using a randomized cross-over design with a 24-h washout period between treatments. The washout period allowed for at least 10 half-lives of either PIM or ODMP to lapse between treatments to assure that the previous dose was eliminated prior to the administration of the second dose. Prior to the study initiation, the dogs were assigned a feeding schedule followed by withholding of food for 10 h to standardize the transit time of the luminal content as much as possible. The dogs were fed a commercial dry dog food during this study, except for not being fed for 10 h before and for 2 h after the administration of pimobendan. The dose of pimobendan and the sampling times used in this study were based on a pilot study in two additional dogs for which a dose of 0.25 mg/kg PR resulted in concentrations that were insufficiently quantifiable to allow a pharmacokinetic analysis (data not shown). As such, for this study, the dogs received pimobendan orally at 0.5 mg/kg to the nearest tablet size (1.25, 2.5, or 5 mg Vetmedin tablet, Boehringer Ingelheim), with the rectal dose being the same (mg) as the oral dose for each dog. Oral dosing was immediately followed by the administration of 5 ml water by syringe over the base of the tongue for complete swallowing. For the rectal administration, pimobendan suspensions were prepared a maximum of 30 min prior to the scheduled administration. The calculated dose of the pimobendan tablet for each animal was finely crushed to assure adequate disintegration. Then, the crushed tablet for each dog was prepared as a solution in 0.9% NaCl (0.9% NaCl, Sagent Pharmaceuticals, Schaumburg, IL, USA) by using two 20-ml Luer Lock syringes connected to a three-way stopcock to yield a pimobendan solution of 1 mg/ml. After mixing the entire suspension, thus the entire dose for each animal and only for that animal, the solution was pulled up into a syringe, and the total dose was administered ~10 to 15 cm into the rectum in a 20-ml syringe, with a 16-in. (41 cm), 10 French red rubber tube (feeding tube and urethral catheter, 10 Fr, Kendall, Tyco Healthcare Group LP, Mansfield, MA, USA). To assure that the entire dose was administered, the tube in which the dose was mixed was washed with a final 5 ml of 0.9% NaCl and this was used to flush the tube, thus assuring that the entire calculated dose was administered to each animal. After the administration, the anus was digitally held closed for ~3 min to prevent the early expulsion of the drug.

### Sample Collection

For all the dogs, a 19 g, 16- or 25-cm central-line catheter (MILACATH 19 g, 25-cm intravenous catheter, item # PI1910; Mila International, Inc., Erlanger, KY, USA) was placed in the saphenous vein for blood sampling, as described elsewhere (16). The catheter was “locked” with unfractionated heparin at a concentration of 200 U/ml. The catheter site was covered with sterile non-adherent dressing and secured with a wrap. An Elizabethan collar was placed on each cat to help prevent self-removal of the catheter. Twelve blood samples were obtained from the catheter immediately before the drug administration (0) and at 5, 10, 15, 30, 45, 60, 120, 240, 360, 600, and 720 min thereafter for each of the two study periods. At 12 h after the final blood sampling was collected following the first dose, the dogs received pimobendan via the alternate route and the study was repeated as detailed above. After the collection of the final sample at 720 min, the IV catheter was removed, and the dogs were released home to their owner. All blood samples were collected into tubes containing lithium heparin (monoject lithium heparin tubes, Fisher Scientific, Itasca, IL, USA) and placed over ice until processing. Plasma was separated within 1 h by centrifugation at 27°C (3,500 × *g*, 10 min) and frozen separately at −70°C until analysis.

### Analytical Method

Plasma PIM and ODMP were quantified using high-performance liquid chromatography (HPLC) with ultraviolet detection as previously reported but with modifications. The HPLC system consisted of a Waters 717 plus auto-sampler, Waters Binary pump 600 controller, and a 2,487 UV–visible detector (Waters Corporation™, Milford, MA, USA) (17). Briefly, separation was achieved with a Gemini C6, Phenyl 110A, 5 μm, 150 × 3.0 mm column (Phenomenex®, Torrance, CA, USA) at 40°C (18). The mobile phase consisted of 0.6% ammonium acetate buffer, pH 3.0, and acetonitrile (VWR®, Radnor, PA, USA), with the flow rate set to 0.8 ml/min (17, 19). For the sample preparation, briefly (6, 17, 18), 1,000 μl of acetonitrile was added to tubes containing 500 μl of plasma. The contents of each tube were mixed vigorously through vortexing and then subjected to centrifugation for 10 min at 1,900 × g at room temperature. The clear supernatant was transferred to a clean glass tube. The supernatant was evaporated to dryness under a gentle stream of nitrogen. The residue was reconstituted with 250 μl of mobile phase, vortexed for 20 s, and then the solution was centrifuged for 10 min. A total of 100 μl was injected into the column. The retention time for PIM was 6.0 min, and for ODMP it was 3.0 min. PIM and ODMP were detected with UV absorbance at 330 nm. The quantitation of PIM and ODMP was based on the standard curves prepared in canine plasma containing known amounts of PIM (Sigma-Aldrich®, St. Louis, MO, USA) and ODMP (Cerilliant® Round Rock, Texas, USA). The standard curve concentrations ranged from 1 to 200 ng/ml for both PIM and ODMP. A standard curve was accepted if the coefficient of determination (*r*^2^) was at least 0.99 and the predicted concentrations were within 10% of the actual concentrations.

The linear correlation coefficient for the PIM and the ODMP standard curves was 0.998. The limit of detection for PIM and ODMP in canine plasma was 0.5 and 1 ng/ml, respectively. The lower limit of quantification for PIM and ODMP in canine plasma was 1 and 2 ng/ml, respectively. The precision (CV %) for PIM in canine plasma at 2, 14, 50, and 76 ng/ml was 2.49, 1.80, 2.74, and 3.90%, respectively. The accuracy (% recovery) for PIM in canine plasma at 2, 14, 50, and 76 ng/ml was 100.10, 103.43, 105.86, and 105.81%, respectively. The precision (CV %) for ODMP in canine plasma at 2, 14, 50, and 76 ng/ml was 4.41, 2.26, 2.39, and 4.23%, respectively. The accuracy (% recovery) for ODMP in canine plasma at 2, 14, 50, and 76 ng/ml was 101.40, 104.75, 103.09, and 104.45%, respectively.

### Pharmacokinetic Analysis

Plasma PIM and ODMP concentration vs. time data were subjected to non-compartmental analysis using computer software (Phoenix WinNonlin, version 7.0, Certera Corp., Princeton, NJ, USA). Area under the concentration-vs.-time curve from zero to the last time point (AUC) was determined using the log-linear trapezoidal method. The actual *C*_max_ occurring at time to maximum concentration (*T*_max_) was recorded. Concentration at 12 h (C12) and at the last time point collected (*C*_min_) was also recorded. The slope of the terminal component of the drug–elimination time curve was based on non-linear regression. Because pimobendan was not given intravenously, the terminal component could not be confirmed to have been eliminated and thus both the terminal rate constant and the corresponding half-lives were reported as disappearance; half-life (*t*_1/2_) was reported as harmonic mean ± pseudo-standard deviation. Furthermore, neither clearance (CL) nor volume of distribution (*V*_d_) could be determined and both are reported as the ratio of either to bioavailability (*V*_d_/F or CL/F). Other parameters included mean residence time and the percent of the AUC that was extrapolated from the terminal component of the curve. The relative bioavailability of PIM and ODMP was estimated by AUC_PR_/AUC_PO_. The metabolite–parent AUC ratio was estimated by AUC_ODMP_/AUC_PIM_.

### Statistical Analysis

Statistical analyses were conducted by a commercially available spreadsheet (Microsoft Office 2010; Microsoft Excel, version 14, Microsoft Corporation, 2010, Redmond, WA, USA). The pharmacokinetic parameters were reported out as mean ± standard deviation. Kolmogorov–Smirnov test was performed to evaluate the normality of parameters. The following comparisons were made between PO and PR routes, using a two-tailed paired *t*-test: *C*_max_, *T*_max_, and AUC. A *t*-test was used to compare the metabolite–parent AUC ratio between PO and PR. Values were considered significantly different at *P* < 0.05.

## Results

Pimobendan was well tolerated in all dogs receiving both routes. The authors did not observe the expulsion of pimobendan solution after the rectal administration. No adverse effects were noted with both PR and PO pimobendan.

**Figure 1:**
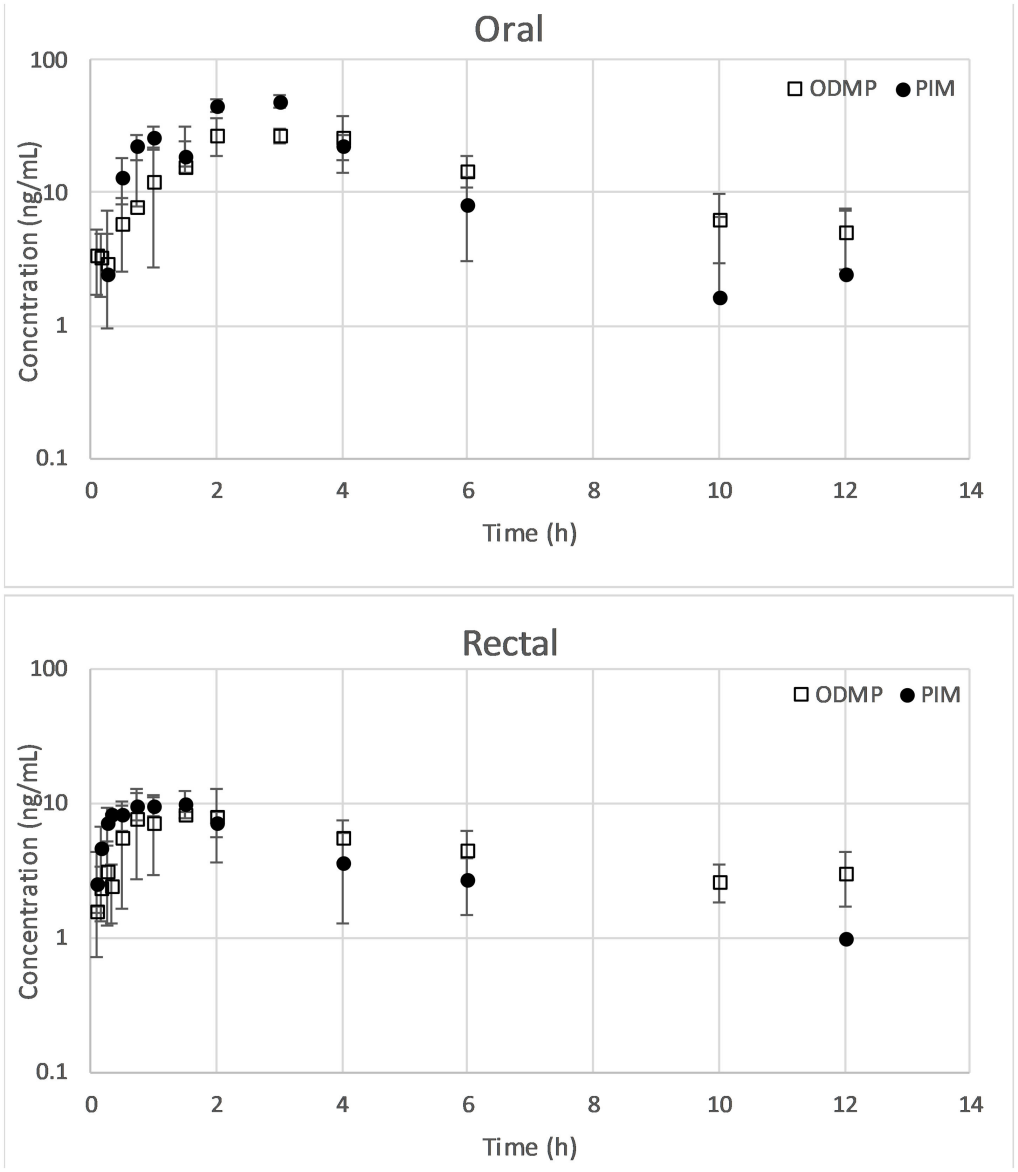
Mean ± SD of concentration–time plots of PIM and ODMP for dogs treated with a single dose of 0.5 mg/kg pimobendan per os (*n* = 7) vs. per rectum (*n* = 8). ODMP, o-desmethyl-pimobendan; PIM, pimobendan.

The mean of the actual doses administered was 0.51 (range 0.5–0.52) mg/kg for PO and 0.5 mg/kg for PR. One dog removed the central line catheter after completion of the PR trial and thus was excluded from the PO trial; the PR data from this dog were retained. Data on the mean ± SD log plasma drug concentration vs. the time plots of PIM and ODMP for either PO or PR are displayed in Figure 1. The mean pharmacokinetic parameters of PIM and ODMP in both routes are summarized in Table 1.

**Table 1:** Summary of pimobendan and O-demethylated-metabolite pharmacokinetic parameters (mean ± SD) for a single dose of pimobendan in dogs.

	**Pimobendan PO**		**Pimobendan PR**		***P*****-value (PO vs. PR)**	
**Parameter**	**PIM**	**ODMP**	**PIM**	**ODMP**	**PIM**	**ODMP**
*C*_max_ (ng/ml)	49.1 ± 28.7	30.9 ± 10.4	10.1 ± 2	8.8 ± 4.8	0.002	0.0001
*C*_min_ (ng/ml)	2.5 ± 1.4	2.8 ± 1.1	1.7 ± 0.9	1.7 ± 0.5	ND	ND
C12 (ng/ml)	2.4 ± 1.6	5.2 ± 2.5	1	3.1 ± 1.3	ND	ND
*T*_max_ (h)	2.1 ± 0.9	3.2 ± 1.6	1 ± 0.4	1.7 ± 1.1	0.01	0.01
*t*_1/2_ (h)	1.8 ± 0.8	5.0 ± 2.7	2.2 ± 0.6	8.3 ± 4.8	>0.05	>0.05
AUC (ng*h/ml)	148.4 ± 71.6	167.8 ± 36.2	31.1 ± 11.9	50.1 ± 19.2	0.03	0.0006
MRT (h)	3.9 ± 1.3	8.3 ± 3.5	3.5 ± 0.9	13.2 ± 8.0	>0.05	>0.05
CL/*F* (ml/h/kg)	0.004 ± 0.002	0.003 ± 0.001	0.012 ± 0.003	0.010 ± 0.005	ND	ND
*V*/*F* (ml/kg)	0.011 ± 0.009	0.022 ± 0.013	0.036 ± 0.002	0.120 ± 0.074	ND	ND
*F* (AUC_PR_/AUC_PO_, %)			25 ± 8	28 ± 6		

*AUC, area under the concentration-vs.-time curve; CL/F, the ratio of clearance to bioavailability; C_max_, maximum plasma concentration; C_min_, concentration at the last time point collected; C12, plasma concentration at 12 h; F, relative bioavailability; MRT, mean residence time; ODMP, o-desmethyl-pimobendan; PIM, pimobendan; PO, per os; PR, per rectum; t_1/2_, disappearance half-life; T_max_, time to maximum concentration; V/F, the ratio of volume of distribution to bioavailability*.

Plasma PIM and ODMP concentrations from pimobendan PR were found to be comparatively low at all timepoints compared to pimobendan PO. Significant differences in *C*_max_, *T*_max_, and AUC of PIM and ODMP were observed between PO and PR (*P* < 0.05; Table 1). Pimobendan PR resulted in a significantly lower *C*_max_ (PIM 10.1 ± 2 ng/ml, ODMP 8.8 ± 4.8 ng/ml) than pimobendan PO (PIM 49.1 ± 28.7 ng/ml, ODMP 30.9 ± 10.4 ng/ml). Pimobendan was more rapidly absorbed *via* PR (*T*_max_ 1 ± 0.4 h) than PO (*T*_max_ 2.1 ± 0.9 h) (*P* = 0.01). The relative bioavailability (relative *F* = AUC_PR_/AUC_PO_) of PIM and ODMP was 25 ± 8 and 28 ± 6, respectively, indicating that AUC_PR_ was significantly lower than AUC_PO_ (*P* < 0.05).

Pimobendan was rapidly converted to ODMP after both PO and PR administrations because ODMP was detected in plasma within minutes (Figure 1). The *t*_1/2_ of PIM was shorter than that of ODMP in both routes. The concentration–time profile of ODMP lagged slightly behind that of PIM in both routes. The AUC_ODMP_ tended to be higher than the AUC_PIM_ in both routes. The metabolite–parent ratio (AUC_ODMP_/AUC_PIM_) was 1.46 ± 68 and 1.34 ± 1.25 for PO and PR, respectively. No statistical difference between PO and PR routes was detected (*P* > 0.05).

## Discussion

To the best of the authors' knowledge, this study is the first to describe the pharmacokinetic profiles of PIM and its active metabolite ODMP following a rectal administration in dogs. In the authors' experiences, dogs presenting with CHF and requiring emergency treatment can be in severe respiratory distress, which may make the oral administration of pimobendan challenging in the United States where tablet is the only form available. The size of pimobendan tablets is relatively larger compared to other tablets, which makes it even more difficult to administer pimobendan PO especially to those patients. Furthermore, there are scenarios when dogs in respiratory distress vomit after medication has been administered PO, which makes the absorption to achieve a therapeutic concentration questionable (20). For those scenarios, pimobendan PR may provide an option for short-term therapy in dogs as their first dose. This study demonstrated that pimobendan PR can achieve a presumed therapeutic plasma concentration of PIM and ODMP in healthy dogs based on the pharmacokinetic profiles provided in the package insert (15). Furthermore, pimobendan PR showed similar metabolite–parent AUC ratio (AUC_ODMP_/AUC_PIM_) compared to its oral administration. Therefore, the findings in this study suggest that the use of pimobendan PR may achieve therapeutic plasma concentrations similar to pimobendan PO.

To determine the pharmacokinetic profile and relative bioavailability of pimobendan PR, this study used a dose that was two times greater (0.5 mg/kg) than that routinely used in oral administration (0.25 mg/kg) (4). Pimobendan decreases the left atrial pressure in a dose dependent manner, and thus the higher dose (0.5 mg/kg) may be potentially beneficial as short-term therapy for dogs with CHF (21, 22). Previous pharmacokinetic studies in dogs suggested that the dosage for rectal administration should be a higher dose than that of oral administration based on the relatively lower bioavailability, *C*_max_, and AUC compared to those of oral administration (9–11, 23, 24). The possible reasons for this include the relatively small surface in the dogs' rectum being available for drug uptake, the presence of feces sequestrating drugs in the rectum at the time of administration, or the possibility of inadvertent expulsion of drugs after administration (12, 13, 24). Similar to previous studies, pimobendan PR showed significantly lower AUC and *C*_max_ compared to those of oral administration (*P* < 0.05). Furthermore, in order to have sufficient data points to determine the relative bioavailability of pimobendan PR, the authors determined that 0.5 mg/kg dosing was necessary to be able to have sufficiently high plasma drug concentrations to allow quantitation. Pimobendan 0.5 mg/kg PR was well tolerated in this study, with no obvious signs of gastrointestinal discomfort or other physiologic changes. This study is not designed to evaluate the potential adverse effects of the long-term administration of pimobendan at a dose of 0.5 mg/kg because the PR route will benefit dogs that received a single dose during initial stabilization.

In this study, the authors determined that 0.9% NaCl was appropriate for drug delivery because of its accessibility in clinical practice in comparison to specifically formulated suppositories. In addition, the dogs in the present study did not receive an enema or manual evacuation of fecal material prior to the PR administration of pimobendan solution. These methods that we used can be followed by any veterinarian with no specialized equipment. The solutions tend to be absorbed more quickly through the rectum, produce effects more rapidly, and may not avoid first-pass metabolism compared to other formula (12, 13, 24). Although faced with the risk of impaired absorption due to the presence of feces, we chose not to manually evacuate feces or perform enema in order to better approximate the conditions in which pimobendan PR will be used in a clinic. This is clinically important as dogs requiring pimobendan PR might have fecal material in the rectum at the time of administration.

This study demonstrated that the rectal administration of pimobendan (0.5 mg/kg, PR) can achieve a plasma concentration of PIM and ODMP in healthy dogs which is higher than those provided in the package insert (15). Provided that the plasma concentrations of PIM and ODMP in the package insert produce the desired hemodynamic effects in dogs, an analysis of the results of our study suggests that a higher dose of pimobendan PR could potentially be an alternative to PO. However, the therapeutic efficacy of the plasma concentrations reported in our study is difficult to interpret, partly because the plasma concentrations associated with the presumed efficacy of PIM and OMDP are not clearly defined in dogs. The authors noticed a disparity among the pharmacokinetic profiles of pimobendan reported by others (1, 6, 21). The key pharmacokinetic parameters of PIM and ODMP from different studies are summarized in Table 2. Pimobendan capsule 0.25 mg/kg resulted in a *C*_max_ of 38.1 ± 18.3 ng/ml (1). In two other studies, both using pimobendan suspensions (but different products), 0.27 mg/kg of a pimobendan suspension produced *C*_max_ of 18.6 ng/ml (6.1–25.3), yet 0.3 mg/kg resulted in a *C*_max_ of 7.3 ± 2.7 ng/ml (6, 21). This disparity may be due to the differences in the design of the experiment (e.g., the dogs that participated in each study and the analytical method) or the effects of different formulations of the drug absorbed. Therefore, the clinical usefulness of pimobendan administered PR in the present study warrants further investigation combined with pharmacodynamic investigation to determine the correlation between plasma concentrations and the cardiovascular effects.

**Table 2:** Data of previous studies investigating the pharmacokinetic properties of pimobendan in dogs [all presented as arithmetic mean ± SD except for Yata et al. presented as (median, range)].

	**Bell et al**. **(****1****)**			**Yata et al**. **(****6****)**		**Package insert**		**Her et al. (this study)**				**Guth et al. (21)**
**Formulation**	**Capsule**	**Solution**	**IV**	**Solution**		**Tablet**		**Tablet**		**Solution**		**Suspension**
**Route**	**PO**		**IV**	**PO**		**PO**		**PO**		**PR**		**PO**
**Dose (mg/kg)**	**0.25**		**0.125**	**0.27**		**0.25**		**0.5**		**0.5**		**0.3**
	**PIM**			**PIM**	**ODMP**	**PIM**	**ODMP**	**PIM**	**ODMP**	**PIM**	**ODMP**	**PIM**
*C*_max_ (ng/ml)	38.1 ± 18.3	39.4 ± 23.4	51.1 ± 28.5	18.6 (6.1–25.3)	16.2 (6.0–22.3)	3.09 ± 0.76	3.66 ± 1.21	49.1 ± 28.7	30.9 ± 10.4	10.1 ± 2	8.8 ± 4.8	7.3 ± 2.7
*T*_max_ (h)	*N*/*A*	*N*/*A*	*N*/*A*	1.1 (0.5–2.0)	1.3 (0.8–2.0)	2	3	2.1 ± 0.9	3.2 ± 1.6	1 ± 0.4	1.7 ± 1.1	3.2 ± 1.3
AUC (ng*h/ml)	*N*/*A*	*N*/*A*	*N*/*A*	27.1 (15.2–44.2)	42.1 (25.1–52.7)	*N*/*A*	*N*/*A*	148.4 ± 71.6	167.8 ± 36.2	31.1 ± 11.9	50.1 ± 19.2	22.5 ± 10.4
*t*_1/2_ (h)	*N*/*A*	*N*/*A*	*N*/*A*	0.9 (0.7–1.1)	1.6 (1.3–1.9)	*N*/*A*	*N*/*A*	1.8 ± 0.8	5.0 ± 2.7	2.2 ± 0.6	8.3 ± 4.8	*N*/*A*
*F*	*N*/*A*	*N*/*A*	*N*/*A*	N/A	N/A	*N*/*A*	*N*/*A*			25 ± 8	28 ± 6	*N*/*A*
LLOQ (ng/ml)	2.5	2.5	2.5	0.5	0.5	*N*/*A*	*N*/*A*	1	2	1	2	*N*/*A*
Analytical method	*LCMS*	*LCMS*	*LCMS*	UHPLC-MS	UHPLC-MS	*N*/*A*	*N*/*A*	HPLC	HPLC	HPLC	HPLC	*N*/*A*

*AUC, area under the concentration vs.-time curve; C_max_, maximum plasma concentration; F, relative bioavailability; LCMS, normal-phase liquid chromatography–mass spectrometry analysis assay; LLOQ, lower limit of quantification; ODMP, o-desmethyl-pimobendan; PIM, pimobendan; PO, per os; PR, per rectum; t_1/2_, disappearance half-life; T_max_, time to maximum concentration; UHPLC-MS, ultra-high-performance liquid chromatography–mass spectrometer assay*.

The notable findings of this PK study include the fact that pimobendan appears to be more rapidly absorbed when it was administered PR than PO based on *T*_max_, which was significantly shorter for PR for both PIM and ODMP (1–1.5 h earlier, respectively) compared to the PO route (*P* = 0.01 for both PIM and ODMP). The more rapid absorption following rectal administration has the potential to be advantageous over oral administration as it reduces the lag time from treatment to effect in dogs with severe respiratory distress. In addition, pimobendan was more rapidly converted into its metabolite ODMP. When administered PO, pimobendan undergoes the first-pass hepatic metabolism via oxidation and gets converted into ODMP (2, 6, 14). It is known that the PDE3 inhibitory action of ODMP is significantly more potent than that of the parent pimobendan (6, 14, 25). Endoh et al. reported that ODMP is 500 times more potent of an inotrope compared to the PIM in isolated canine ventricular muscle (14). Therefore, ODMP is active and may have an important contribution to the hemodynamic effects of pimobendan. However, there is sparse data regarding the pharmacokinetic profile of ODMP in dogs (26). The manufacturer's package insert reported that pimobendan 0.25 mg/kg PO resulted in an ODMP *C*_max_ of 3.66 ± 1.21 with *T*_max_ at 2 h (15). Yata et al. reported that when nonaqueous solution of pimobendan 0.27 mg/kg was administered PO, pimobendan was rapidly converted to ODMP and the systemic exposure was greater for ODMP than for PIM due to slower elimination (6). These findings are similar to the results in our study.

An important aspect of rectal administration is the possibility of partial avoidance of first-pass hepatic metabolism (12, 13). This is dependent on how far caudally the drug is deposited within the rectum (24). If the drug is administered in the lower part of the rectum, the drug absorbed may bypass the liver and be delivered directly to the systemic circulation, whereas the drug absorbed in the proximal portion of the rectum is drained by the portal vein and still undergoes first-pass metabolism (13). First-pass metabolism becomes more clinically important if the parent compound is metabolized to an active metabolite, as is the case with pimobendan. An analysis of our findings suggests that pimobendan PR does not appreciably avoid first-pass metabolism in dogs. The metabolite–parent AUC ratio (AUC_ODMP_/AUC_PIM_) following pimobendan PR was similar to that of pimobendan PO. However, the dogs' rectums are ~5-cm long, and 2.5 cm of the rectum drains into the systemic circulation (24). Furthermore, it is likely that the length of the rectum will vary significantly depending on the breed and the size of each individual. Therefore, it is highly likely that pimobendan administered PR was absorbed to the portions of the rectum that do not bypass the portal system and thus PIM was converted to ODMP in a similar magnitude following rectal administration. Therefore, the findings in our study showed the potential benefits of pimobendan PR based on more rapid absorption compared to PO and similar magnitude of first-pass metabolism. Further studies will need to include more animals with sampling at more frequent time intervals to more fully characterize the pharmacokinetic profile after rectal administration in dogs.

A limitation to this study includes the lack of injectable pimobendan in addition to oral and rectal dosing. Unfortunately, pimobendan is not available for intravenous administration in the United States and the manufacturer does not anticipate pursuing an approval of any other pimobendan. Bioavailability is most accurately determined by comparing the AUC measurements from intravenous and non-intravenous administration of a drug. Although it may not present the most precise pharmacokinetics data, this study provides a powerful insight to the pharmacokinetic profile of rectally administered pimobendan. The result of this study shows the efficacy of the rectal route to complement the oral administration of the tablet form in medical emergencies. An additional limitation to this study is the fact that healthy dogs were used. The dogs used in this study were considered healthy based on history, physical examination, and clinicopathological findings. Using healthy animals is often necessary in such studies. On the other hand, when rectal dosing is used in the clinical setting, this route will commonly be administered to dogs with CHF and with decreased cardiac output leading to end-organ hypoperfusion. It is possible that due to the reduced cardiac output, age, concurrent treatments, or other comorbidities, the bioavailability, metabolism, or excretion of pimobendan could be altered in dogs who will receive benefit from this route. Another concern is that the dogs also may expel an unmeasurable amount of the drug following rectal administration. However, it is important to note that the expulsion of medication administered PR can make it difficult for the practitioner to determine how much drug has been absorbed. The last limitation of this study is that the final concentration of the pimobendan solution prior to administration was not measured. The methods used to prepare the pimobendan solution in this study were designed to mimic techniques that have been used in clinics. In the pilot study, an analysis of pimobendan solution prior to administration showed that the concentration of pimobendan solution administered was consistently 30% less than the intended concentration. Therefore, the actual dose of pimobendan in the administered solution in the pivotal study could be lower than the intended dose. As such, the bioavailability reported in the current study can be lower than the one from expected concentration.

## Conclusion

In conclusion, the bioavailability of pimobendan following rectal administration was lower compared to that of oral administration. We conclude that the rectal administration of a single-dose (0.5 mg/kg) pimobendan achieved presumably therapeutic plasma concentration in dogs based on the package insert. Given the rapid absorption to achieve maximum concentration in conjunction with the metabolite–parent AUC ratio similar to oral administration, the rectal administration of pimobendan may be a suitable option for the immediate stabilization of dogs with CHF. Additional studies need to be performed to determine the recommended therapeutic target range of plasma concentration of PIM and ODMP in dogs. Further studies are warranted to describe the pharmacokinetics of pimobendan following repeated dosing in conjunction with pharmacodynamics investigation for dosing recommendation for this route.

## Data Availability Statement

The datasets generated for this study are available on request to the corresponding author.

## Ethics Statement

The animal study was reviewed and approved by Auburn University Institutional Animal Care and Use Committee. Written informed consent was obtained from the owners for the participation of their animals in this study.

## Author Contributions

JH contributed to study design, sample collection, data analysis, and manuscript preparation. KK read and approved the final manuscript, contributed to study design, and sample collection. RW contributed to study design and approved the final manuscript. CC-E contributed to manuscript preparation, sample collection, and data analysis. LB contributed to study design and read and approved the final manuscript. DB contributed to study design, sample collection, data analysis, and manuscript review and approved the final manuscript. All authors contributed to the article and approved the submitted version.

## Conflict of Interest

The authors declare that the research was conducted in the absence of any commercial or financial relationships that could be construed as a potential conflict of interest.

## Abbreviations

ACVIM: American College of Veterinary Internal Medicine
AUC: area under the concentration-vs. -time curve
CHF: congestive heart failure
CL/F: the ratio of clearance to bioavailability
*C*_max_: maximum plasma concentration
C12: plasma concentration at 12 h
*F*: relative bioavailability
HPLC: high-performance liquid chromatography
LLOQ: the lower limit of quantification
MRT: mean residence time
ODMP: o-desmethyl-pimobendan
PDE3: phosphodiesterase 3
PIM: pimobendan
PO: per os
PR: per rectum
*t*_1/2_: disappearance half-life
*T*_max_: time to maximum concentration
*V*/*F*: the ratio of volume of distribution to bioavailability.
